# Early Evaluation of Myeloperoxidase and Delta Neutrophil Indices Is Similar to 48 h Sequential Organ Failure Assessment Score for Predicting Multiple Organ Failure After Trauma

**DOI:** 10.3390/jcm14103447

**Published:** 2025-05-15

**Authors:** Woo Jin Jung, Hye Sim Kim, Kyoung Chul Cha, Young-Il Roh, Gyo Jin An, Yong Sung Cha, Hyun Kim, Kang Hyun Lee, Sung Oh Hwang, Oh Hyun Kim

**Affiliations:** 1 Department of Emergency Medicine, Yonsei University Wonju College of Medicine, Wonju 26426, Republic of Korea; wjjung21c@yonsei.ac.kr (W.J.J.); chaemp@yonsei.ac.kr (K.C.C.); md.youngilroh@yonsei.ac.kr (Y.-I.R.); minstrel@yonsei.ac.kr (G.J.A.); emyscha@yonsei.ac.kr (Y.S.C.); khyun@yonsei.ac.kr (H.K.); ed119@yonsei.ac.kr (K.H.L.); shwang@yonsei.ac.kr (S.O.H.); 2 Center of Biomedical Data Science, Yonsei University Wonju College of Medicine, Wonju 26493, Republic of Korea; sam0246@naver.com

**Keywords:** multiple organ failure, injuries, injury severity score

## Abstract

**Background/Objectives**: Multiple organ failure is the main cause of mortality in severely injured patients who survive the early post-trauma phase. Myeloperoxidase and delta neutrophil indices may serve useful markers for the early diagnosis of an inflammatory condition. We aimed to ascertain the use of these indices for predicting multiple organ failure after a major trauma. **Methods**: A retrospective study was performed based on a level I trauma center database that included trauma patients with an injury severity score of >15 points. Organ function was evaluated according to the sequential organ failure assessment score within at least 48 h of admission and the myeloperoxidase and delta neutrophil indices, which were measured every morning. **Results**: A total of 96 patients were included in this study. Compared with the non-multiple-organ-failure group, the multiple organ failure group had similar myeloperoxidase indices but a significantly higher delta neutrophil index. Multivariate logistic regression analysis revealed no significant difference in the predictive power among the post-trauma multiple organ failure models that included various factors, although model 7, which combined the sequential organ failure assessment score and the myeloperoxidase and delta neutrophil indices, tended to have the maximum predictive power. **Conclusions**: Early delta neutrophil index (DNI) values and the composite model incorporating SOFA, absolute MPXI, and DNI each demonstrated moderate ability to predict multiple organ failure after major trauma. Prospective multicenter studies that include granular treatment variables are warranted to validate these biomarkers and to explore whether their incorporation into real-time decision tools can improve outcomes.

## 1. Introduction

Multiple organ failure (MOF) is a major cause of mortality in critical patients and has been reported as a major complication after blunt trauma in recent decades [1]. Post-trauma MOF causes 51% to 61% of late trauma deaths [2] and is usually preceded by prolonged hemorrhagic shock. Post-trauma MOF is the result of an uncontrolled systemic inflammatory response and has been a major cause of late trauma death in the past [3]. Despite changes in the epidemiology and gradual improvements in mortality, MOF remains the leading cause of late post-trauma mortality and extended stay in the intensive care unit (ICU) [4]. Therefore, more accurate prediction of MOF might influence the physician’s therapeutic plan and the prognosis of severely injured patients.

Post-trauma hyperinflammation is characterized by local and systemic release of proinflammatory cytokines, metabolites, and acute-phase proteins that lead to systemic inflammatory response syndrome (SIRS) [5,6]. Although the use of several cytokine biomarkers for the prediction of post-trauma MOF has been studied, the cytokine assay method is time-consuming and costly [7,8,9]. Recently, some researchers have reported the myeloperoxidase index (MPXI) and delta neutrophil index (DNI) as biomarkers of systemic inflammation, sepsis, and MOF [10,11,12,13]. Myeloperoxidase (MPO) activates the synthesis of hypochlorous acid from hydrogen peroxide and chloride ions, and hypochlorous acid plays a role in the defense against bacteria, fungi, and viruses [14,15]. The DNI, which reflects the fraction of circulating immature granulocytes, has been associated with increased mortality in patients with systemic inflammation [16] and increases during inflammatory and infection process. MPXI and DNI can be measured early and automatically calculated along with complete blood count, with no additional cost.

The predictive utility of MPXI and DNI has been reported in SIRS, sepsis, and MOF due to infection, but only a few reports are available for post-trauma MOF [17]. Therefore, this study aimed to evaluate the changes in the MPXI and DNI among trauma patients and to investigate the utility of early MPXI and DNI as predictive biomarkers of post-trauma MOF. We hypothesized that the early values of MPXI or DNI were significantly associated with MOF progression among injured trauma patients.

## 2. Materials and Methods

### 2.1. Study Setting and Enrolled Patients

**Figure 1:**
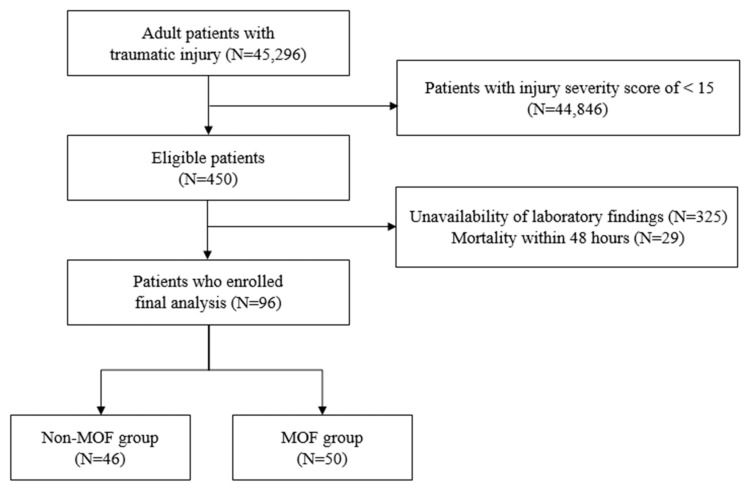
Diagram of patient enrollment.

A retrospective observational study was performed at Wonju Severance Christian Hospital from January 2018 to March 2022. This study was approved by the Institutional Review Board of Wonju Severance Christian Hospital (CR317354). Because the study was a retrospective review, the need for informed consent was waived by the Institutional Review Board. This hospital had been designated a regional emergency medical center and a level I trauma center, where about 43,000 patients are seen annually at the emergency department (ED). During the study period, 164,989 patients visited the ED and trauma center. Of these patients, 45,296 were enrolled in a trauma registry; we enrolled severely injured trauma patients who met an injury severity score (ISS) of >15 and had consecutive confirmation of MPXI and DNI within the initial 48 h (Figure 1). Early measurement of MPXI and DNI was defined as values within 0 to 48 h of admission. Then, if the patients underwent additional complete blood counts, the values of MPXI and DNI were confirmed on consecutive days for two weeks (Figures S1 and S2). The exclusion criteria were patients under the age of 18 years, those who were diagnosed with non-traumatic disease, those who were treated only in the general ward, and those who died within the first 48 h after admission.

### 2.2. Measurement of Variables

To check the presence of post-trauma MOF, we reviewed the medical records of total patients from ED admission to hospital discharge. The occurrence of MOF was defined as simultaneous failure of two or more organs including respiratory, blood coagulation, hepatobiliary, cardiovascular, central nervous system, or kidney damage [18]. Moreover, the sequential organ failure assessment (SOFA) score, which is commonly used as an organ dysfunction score, was confirmed as the maximum value calculated 48 h after initial ED arrival (Table 1) [19]. The following parameters about baseline characteristics were collected: age, sex, ISS score, Glasgow coma scale, Rotterdam CT score for evaluate damaged brain [20], injury mechanism, SOFA score within 48 h of ED admission, length of ICU admission, the overall hospital length of stay, application of mechanical ventilation, and vasopressor use. In addition, the Denver MOF score was also calculated for relative comparison with the SOFA score [21]. The consecutive daily values of MPXI and DNI were collected within 48 h of ED admission, and cases with missing values were excluded. The overall injury severity was classified by the established ISS, based on the abbreviated injury acale [5]. MPXI and DNI were measured in blood samples taken during hospital admission from the ED to the ICU. We also investigated the amount of packed red blood cells and fresh frozen plasma transfused in the first 48 h and measured serum levels of blood lactate and base excess during the same period.

A specific type of automatic cell analyzer (ADVIA 120/2120; Siemens, Tarrytown, NY, USA) was used to measure serum MPXI and DNI. This flow-cytometry-based hematologic analyzer used two independent methods of measuring the white blood cell count or the leukocyte subfraction assay: by a cytochemical reaction using the MPO channel or based on reflected light beam measurements using the lobularity/nuclear density channel. DNI was calculated using the following formula [22,23]:

DNI = leukocyte subfraction assayed using the MPO channel − leukocyte subfraction assayed using the nuclear lobularity channel.

Using the above-mentioned blood autoanalyzer that used 4-chloro-1-naphthol, which is a substrate for MPO in granulocytes, the mean MPXI was calculated based on the black precipitates that formed in the granulocytes. When the stained WBCs passed to the flow cell, light scatter (*y*-axis) and absorbance (*x*-axis) were measured using a tungsten–halogen light source; the deviation from the mean *x*-axis neutrophil values of an archetypal population defined the MPXI [24]. The ADVIA-2120 expresses MPXI as a signed deviation (±) from the reference neutrophil population. Prior work showed markedly negative MPXI in bacterial sepsis and markedly positive values in non-septic bacterial infections, with both extremes being clinically relevant [12]. To avoid algebraic cancellation and to capture the magnitude of MPO dysregulation, we prospectively analyzed the absolute value of MPXI as a continuous predictor.

### 2.3. Statistical Analysis

The data were analyzed using the Statistical Package for the Social Sciences (SPSS; version 22; IBM Inc., Somers, NY, USA) and the R Statistical Package (version 3.5.1; Institute for Statistics and Mathematics, Vienna, Austria; www.R-project.org accessed on 8 March 2023). Categorical variables are presented as frequencies and percentages. To compare continuous variables, an independent two-sample *t*-test or nonparametric Mann–Whitney U test was used, based on the normality assumptions from the Kolmogorov–Smirnov test. The values were presented as the mean ± standard deviation or as the median and 25th to 75th percentile interquartile range (IQR). Variables showing a Gaussian distribution (GCS, ISS, maximum SOFA at 48 h, Rotterdam CT score, PT-INR, and 24 h PRBC transfusion volume) are summarized as the mean ± standard deviation and compared with an independent-samples t-test. All remaining continuous variables demonstrated non-Gaussian distributions; these are reported as the median and interquartile range (IQR) and compared with the Mann–Whitney U test. The significance level was set at a *p* value < 0.05.

A linear mixed model was used to verify the difference between the groups according to the time points of the repeatedly measured MPXI and DNI. Multiple logistic regression was used to evaluate the relative prediction effects of MPXI and DNI in differentiating between the MOF and non-MOF groups. The area under the receiver operating characteristic curve (AUC) was used to assess the predictive powers of MPXI and DNI for MOF. The AUC comparison was presented using the nonparametric approach. The significance level was set at a *p* value of <0.05.

To determine whether MPXI and DNI were useful predictors of post-trauma MOF, we performed logistic regression analyses, using several models that included age and sex with other varying independent variables, such as ISS for the baseline model. Other parameters used were ISS and maximum SOFA score within 48 h of admission for model 1; ISS and DNI for model 2; ISS and MPXI for model 3; ISS, MPXI, and DNI for model 4; ISS, maximum SOFA score within 48 h of admission, and DNI for model 5; ISS, maximum SOFA score within 48 h of admission, and MPXI for model 6; and ISS, maximum SOFA score within 48 h of admission, MPXI, and DNI for model 7.

Unlike the DNI, which was represented as a value, MPXI was represented as either positive or negative. Therefore, we conducted the analyses by hypothesizing that the absolute values of the MPXI increased the risk of post-trauma MOF. Finally, we selected the maximum values of MPXI and DNI within 48 h and compared the predictive risk for post-trauma MOF among the models.

## 3. Results

### 3.1. Characteristics of Patients

Of the 450 patients, 96 patients who had complete results of complete blood count, including MPXI and DNI, for 48 h were included as the final study subjects (Figure 1). The baseline characteristics of the enrolled patients are shown in Table 2. There were 74 men (77.1%), and the median age was 52 years (IQR, 35.0–69.5 years). There were 50 patients (52.1%) in the non-MOF group and 46 patients (47.9%) in the MOF group. The most common cause of injury was motor vehicular accidents (63.5%), followed by falls (21.9%). The median length of ICU stay and hospital stay were 23 days and 46 days, respectively.

The non-MOF and MOF groups had no significant differences in initial systolic blood pressure, diastolic blood pressure, and body temperature, as well as in the laboratory values of hemoglobin, platelet count, PT-INR, lactate, base excess, and C-reactive protein. The mean ISS did not significantly differ between the non-MOF group and the MOF group. The median hospital stay was similar between the two groups, but the length of ICU stay in the MOF group was longer than in the non-MOF group. The MOF group had transfused a similar amount of packed red blood cells to the non-MOF group within the initial 48 h. However, the MOF group had a significantly higher amount of fresh frozen plasma transfusion within 48 h and a higher mortality rate compared with the non-MOF group (Table 2). Invasive mechanical ventilation and vasopressor therapy were both used markedly more often in patients who developed MOF than in those who did not. There was no significant difference in the types of surgery between the two groups, and 33 patients (34.4%) received only conservative care without surgery or interventional treatment (Table 3).

### 3.2. Myeloperoxidase and Delta Neutrophil Indices

**Figure 2:**
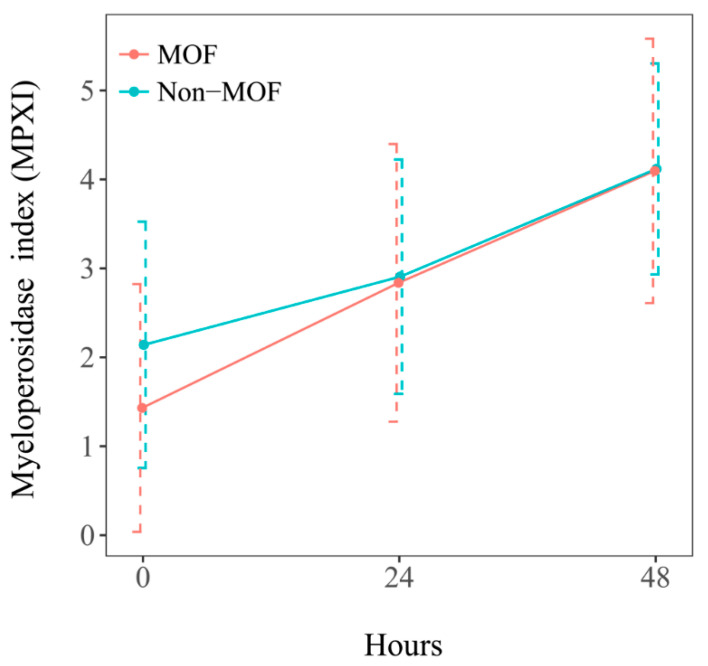
Serum MPXI in patients with severe trauma, according to the presence of multiple organ failure. Comparison of the mean values of consecutive serum myeloperoxidase index (MPXI) for 48 h in severe trauma patients between those with multiple organ failure (MOF) and those without multiple organ failure (non-MOF).

**Figure 3:**
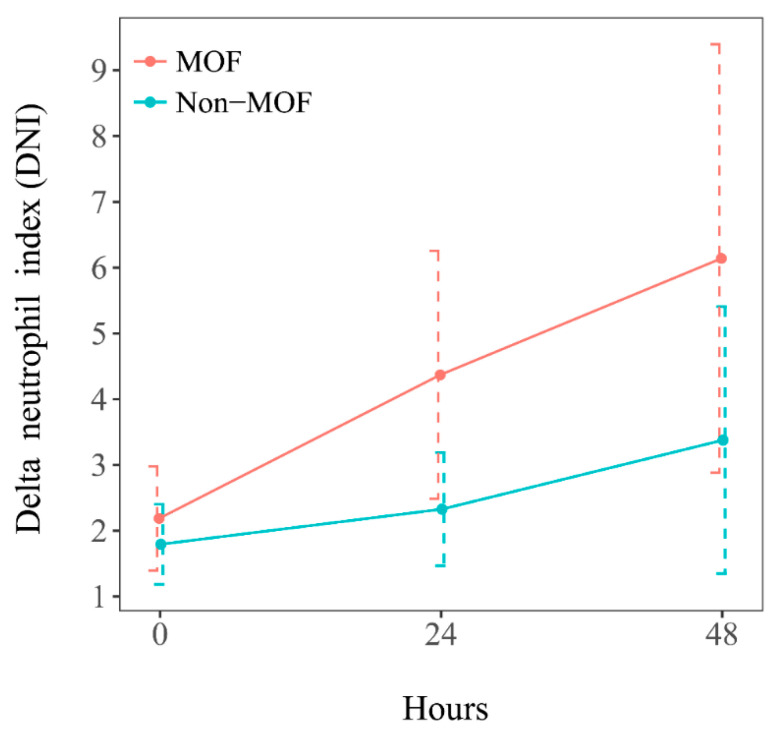
Serum DNI in patients with severe trauma, according to the presence of multiple organ failure. Comparison of the mean values of consecutive serum delta neutrophil index (DNI) for 48 h in severe trauma patients between those with multiple organ failure (MOF) and those without multiple organ failure (non-MOF).

The mean values within the 48 h consecutive measurements of MPXI (normal range, −2 to 2) and DNI (normal range, 0 to 2) were calculated. On the admission day, the mean MPXI of the MOF group was 1.43 ± 4.67, which was lower than the 2.14 ± 4.85 of the non-MOF group. On the first and second day after admission, the mean MPXI of the MOF group was 2.84 ± 5.24 and 4.10 ± 4.98, which was similar to the 2.91 ± 4.61 and 4.12 ± 4.15 in the non-MOF group (Figure 2). On the admission day, the mean DNI of the MOF group was 2.19 ± 2.66, which was higher than the 1.79 ± 2.13 in the non-MOF group. On the first and second day after admission, the mean DNI of the MOF group was 4.37 ± 6.32 and 6.14 ± 10.93, which was higher than the 2.33 ± 3.01 and 3.38 ± 7.10 in the non-MOF group (Figure 3). There was a statistically significant difference in MPXI between time points (*p <* 0.001), but the difference in MPXI between groups was not statistically significant (*p <* 0.524), and the MPXI between groups according to time point was also not statistically significant (*p <* 0.618). There was a statistically significant difference in DNI between time points (*p <* 0.001), but the difference in DNI between groups was not statistically significant (*p <* 0.380). However, the DNI between groups according to time point was analyzed to be borderline (*p <* 0.075). In the MOF group, the DNI rose from 2.19% on admission to 6.14% at 48 h, exceeding the 5% threshold reported in previous trauma studies [17].

### 3.3. Comparative Analysis of Predictive Factors Related to Multiple Organ Failure

**Figure 4:**
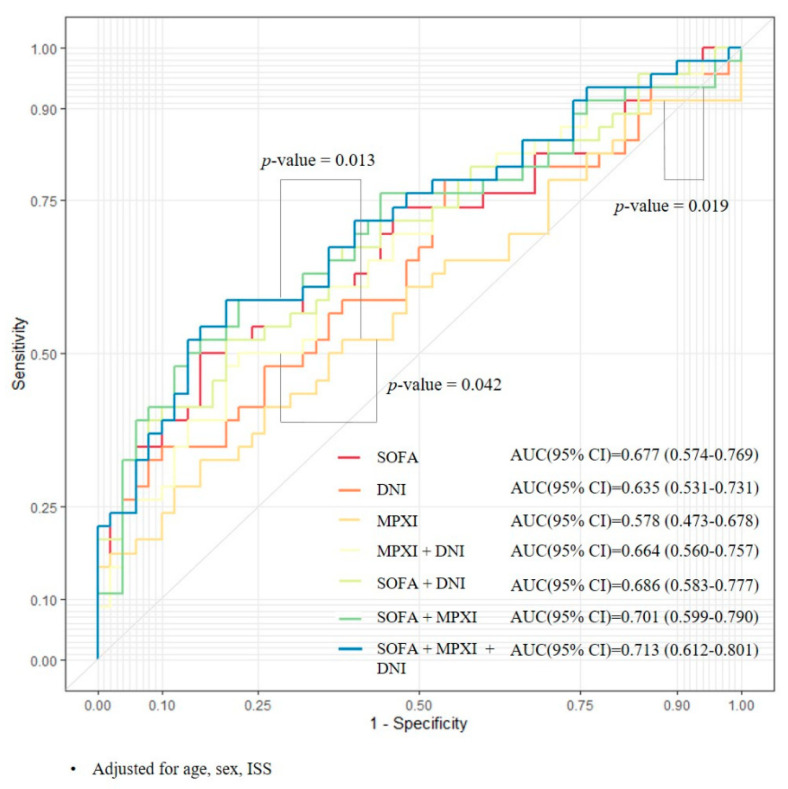
Comparison of the prediction models for multiple organ failure. Comparison of the prediction models, which include the maximum values of SOFA score, DNI, and MPXI, for the probability of post-trauma multiple organ failure within 48 h of admission. SOFA: sequential organ failure assessment; DNI: delta neutrophil index; MPXI: myeloperoxidase index.

Logistic regression analysis with receiver operating characteristic curve analyses revealed that the Harrell’s C-statistics of model 1, which included maximum SOFA within 48 h (AUC 0.677, 95% CI 0.574–0.796), was not significantly superior to model 2, which included DNI (AUC 0.635, 95% CI 0.531–0.731), and model 3, which included MPXI (AUC 0.578, 95% CI 0.473–0.678). Moreover, Harrell’s C-statistics of model 4, which included MPXI and DNI (AUC 0.664, 95% CI 0.560–0.757) did not reveal superior prediction for post-trauma MOF to model 1. Thus, in each model predicting post-trauma MOF, there was no significant difference or superiority with other models compared to model 1 (Figure 4).

## 4. Discussion

The present study explored the usefulness of the myeloperoxidase index (MPXI) and delta neutrophil index (DNI) as early predictors of multiple organ failure (MOF) after severe trauma. Although early DNI exhibited only a modest association with subsequent MOF, its discriminatory ability was comparable to that of the 48 h sequential organ failure assessment (SOFA) score, highlighting both the complex pathophysiology of MOF and the inherent limitations of relying on single biomarkers in the trauma setting.

The etiology of MOF is complicated and may vary between cases, depending on factors related to the patient, injury, and treatment. In injured patients, MOF develops after hemorrhagic shock, resuscitation, and early SIRS. A devastating insult can trigger severe SIRS or subsequent MOF; this situation can be seen as a one-hit model of MOF [2]. A less serious insult with hypotension and reperfusion can trigger a generalized proinflammatory reaction. This secondary insult may cause uncontrolled inflammation and end-organ damage and can be seen as a two-hit model of MOF. This two-hit mechanism includes sepsis, mechanical ventilation, blood transfusion, operation of long bones, secondary surgery, and fat embolism.

Although not statistically significant, the patients who developed MOF in this study had relatively low systolic and diastolic blood pressure, a lower hemoglobin level, and a high amount of transfused packed red blood cells. Moreover, the MOF group received a statistically greater amount of fresh frozen plasma than the non-MOF group. Based on these results of this study population, we thought that the physiological state of the trauma patients who progressed to MOF may have been related to the inflammatory response during the treatment course, either by the one-hit or two-hit mechanism. Prior studies highlight the role of neutrophil activation and immature granulocyte release (reflected by DNI) in amplifying organ damage. While a DNI ≥ 5% has prognostic value in sepsis, its utility in trauma-induced MOF is less established, aligning with our findings of only modest predictive accuracy (AUC 0.635) [12,25].

SIRS may be induced by various factors, including infection, trauma, pancreatitis, burns, and others [26]. There had been some reports on the association of MPXI and DNI with SIRS and sepsis [10,12,13,24]. However, the relevance and predictive ability of MPXI and DNI in patients with post-trauma MOF had not previously been reported. We assessed and compared the predictive ability for post-trauma MOF between early MPXI or DNI and the maximum SOFA score within 48 h of admission. After traumatic injury, we hypothesized that the MPXI and DNI would have higher values in patients with progressing MOF than in those without progressing MOF. The levels of mean MPXI were not significantly different in both groups, but we revealed a significantly higher level of mean DNI in the MOF group than the level of mean DNI in the non-MOF group.

A DNI of 0–2% is generally considered normal, whereas Trauma-ICU data indicate that a DNI ≥ 5% predicts multiple organ dysfunction syndrome and 30-day mortality. In our study, the MOF group exceeded this 5% cut-off within 48 h of injury, reflecting emergency myelopoiesis and pronounced systemic inflammation. A rapidly rising DNI may therefore serve as an early warning signal, prompting timely intensification of infection control and organ support strategies.

Next, we evaluated the predictive powers of MPXI and DNI for post-trauma MOF; however, no clinically useful single value of the 48 h SOFA score, MPXI, or DNI reliably distinguished patients who developed MOF from those who did not. In our cohort, model 7, which included age, sex, ISS, SOFA, MPXI, and DNI, yielded the numerically highest AUC, yet was not statistically superior to simpler models. Although the difference in the predictive power among the models was not statistically significant, these results suggested that the use of multiple factors, including MPXI and DNI, rather than a single factor, would be appropriate to predict post-trauma MOF. ROC comparisons showed that the area under receiver operating characteristic curve values were low even when using multiple parameters. The parameters used here are considered to have low predictive power. However, it is noteworthy that at least the DNI has a similar predictive power to SOFA. Since this study was limited to predicting MOF at an early point in time, we suggest it will be necessary to research other models. Moreover, the modest accuracy observed here may also explain the discrepancy between our findings and earlier studies that reported a strong association between elevated DNI and MOF in mixed ICU cohorts. Whereas we defined MOF using the SOFA score, other investigations relied on the Denver or Marshall scores—systems that assign greater weight to respiratory and cardiovascular dysfunction and thus emphasize different organ systems [21,27]. Such heterogeneity hinders direct comparisons across studies and reinforces the need for consensus MOF criteria.

There have been various studies on the use of biomarkers for the prediction of post-trauma MOF. Measurement of circulating inflammatory cytokines in the early post-trauma phase have been shown to identify patients with progressing MOF. After major traumatic injuries, significantly elevated levels of multiple cytokines and chemokines, such as IL-1RA, IL-6, and IL-10, were recorded in blood samples obtained from injured patients who subsequently developed MOF [28]. Nydam et al. [29] reported that early post-trauma thrombocytopenia was an independent risk factor for MOF, death, and other complications. However, these previous studies that predicted MOF by measurements of existing cytokines or thrombocytopenia were limited to estimating the progression to early-phase MOF after trauma. In addition, we thought that measuring these biomarkers would be ineffective for clinical use in terms of time, effort, and cost. Moreover, in the previous literature, it has been reported that trauma is associated with secretion of self-damage-associated molecular proteins, exposure of the traumatized part of the body to non-self-pattern-associated molecular proteins, and activation of various surface inflammatory receptors [30,31]. Therefore, predicting post-traumatic prognosis with a single marker may be unreasonable.

This study has several limitations that limit interpretation of its findings. First, the small, single-center cohort (n = 96) provides limited statistical power, making it unlikely to detect modest differences in AUC (<0.10) among competing predictive models and restricting generalizability. Second, its retrospective design excluded patients who died within 48 h of admission, a criterion that probably underestimates the true incidence of MOF in severe trauma victims and introduces selection bias; furthermore, key treatment variables—such as fluid balance and transfusion ratios—were unavailable, leaving residual confounding unaddressed. Third, reliance on the SOFA score to define MOF hampers comparison with studies that use the Denver, Marshall, or other scoring systems, because SOFA contains subjective elements (e.g., Glasgow Coma Scale) and omits adrenal dysfunction, thereby contributing to definition heterogeneity across the literature. Fourth, while DNI was measured serially, MPXI’s biphasic behavior (negative in bacterial sepsis vs. positive in sterile inflammation) warrants further investigation in trauma-specific contexts. Finally, none of the evaluated models—including the most comprehensive model combining age, sex, ISS, SOFA, MPXI, and DNI—achieved statistically superior discrimination, so the observed trends remain hypothesis-generating and should be validated in larger, prospective, multicenter studies that incorporate harmonized MOF definitions and serial biomarker assessments.

## 5. Conclusions

Early delta neutrophil index (DNI) values and the composite model incorporating SOFA, absolute MPXI, and DNI each demonstrated moderate ability to predict multiple organ failure after major trauma. While the discriminatory power did not differ statistically between models, the ease of obtaining DNI and MPXI from a routine CBC supports its bedside applicability. Prospective multicenter studies that include granular treatment variables are warranted to validate these biomarkers and to explore whether their incorporation into real-time decision tools can improve outcomes.

## Figures and Tables

**Table 1:** The SOFA score.

	SOFA				
Variables	0	1	2	3	4
Respiration PaO_2_/FiO_2_	>400	≤400	≤300	≤200 with respiratory support	≤100 with respiratory support
Coagulation Platelet (×10^9^/L)	>150	≤150	≤100	≤50	≤20
Liver Total bilirubin (mg/dL)	<1.2	1.2–1.9	2.0–5.9	6.0–11.9	≥12.0
Cardiovascular Hypotension	No Hypotension	MAP < 70 mmHg	Dopamine ≤ 5 or dobutamine (any dose)	Dopamine > 5 or epinephrine ≤ 0.1 or norepinephrine ≤ 0.1	Dopamine > 15 or epinephrine > 0.1 or norepinephrine > 0.1
Central Nervous System Glasgow coma scale	15	13–14	10–12	6–9	<6
Renal Creatinine (mg/dL)	<1.2	1.2–1.9	2.0–3.4	3.5–4.9	≥5.0

SOFA, sequential organ failure assessment; MAP, mean arterial pressure.

**Table 2:** Baseline characteristics of 96 patients with traumatic injury.

	Total (n = 96)	Non-MOF Group (n = 50)	MOF Group (n = 46)	*p* Value
Male (sex)	74 (77.1%)	39 (78.0%)	35 (76.0%)	1.000
Age (years)	52.0 (35.0–69.5)	48.5 (37.5–63.7)	55.5 (27.0–73.2)	0.538
Systolic blood pressure (mmHg)	126 (95–146)	120 (74–142)	96 (77–131)	0.072
Diastolic blood pressure (mmHg)	72 (60–89)	64 (45–80)	61 (46–77)	0.139
Pulse rate (beats/minute)	96 (75–112)	97 (82–111)	101 (74–119)	0.887
Body temperature (°C)	36.0 (35.6–36.4)	36.0 (35.6–36.4)	36.0 (35.1–36.2)	0.221
Glasgow coma scale	11.6 ± 3.8	12.0 ± 3.7	11.2 ± 3.9	0.306
ISS	24.5 ± 8.8	22.9 ± 5.9	26.3 ± 10.9	0.065
AIS1	3 (1–4)	3 (0–4)	3 (2–4)	0.342
AIS2	0 (0–1)	0 (0–1)	0 (0–2)	0.112
AIS3	3 (0–3)	3 (0–3)	3 (1–3)	0.620
AIS4	2 (0–2)	1 (0–2)	2 (0–3)	0.466
AIS5	0 (0–2)	2 (0–2)	0 (0–2)	0.316
AIS6	0 (0–0)	0 (0–0)	0 (0–0)	0.330
SOFA *	5.7 ± 3.5	4.7 ± 2.7	6.8 ± 3.9	0.004
Denver MOF score	1 (0–1)	1 (0–1)	1.5 (0–4)	<0.001
Rotterdam CT score	2.9 ± 1.2	2.8 ± 1.2	2.9 ± 1.1	0.637
Injury mechanism				0.374
Motor vehicle accident	61 (63.5%)	33 (66.0%)	28 (60.8%)	
Falls	21 (21.9%)	10 (20.0%)	11 (23.9%)	
Slip down	6 (6.3%)	3 (6.0%)	3 (6.5%)	
Blunt injury	4 (4.2%)	3 (6.0%)	1 (2.2%)	
Penetrating injury	1 (1.0%)	0 (0%)	1 (2.2%)	
Others	3 (3.1%)	1 (2.0%)	2 (4.4%)	
Laboratory findings				
Hemoglobin	12.8 (10.4–14.2)	13.3 (10.4–14.6)	12.7 (10.4–13.5)	0.364
Platelets	229 (170–283)	235 (167–295)	228 (170–270)	0.697
PT INR ^†^	1.08 ± 0.25	1.0 ± 0.2	1.1 ± 0.2	0.463
C-reactive protein	0.46 (0.29–1.08)	0.71 (0.39–1.48)	0.35 (0.06–0.63)	0.056
Worst lactate, 0–12 h	2.57 (1.79–3.44)	2.68 (1.72–3.61)	2.48 (1.94–3.35)	0.659
Worst lactate, 12–24 h	2.57 (1.70–3.44)	2.68 (1.72–3.59)	2.48 (1.70–3.35)	0.575
Worst base excess 0–12 h	−7.8 (−10.6–−5.1)	−7.7 (−10.6–−4.3)	−8.2 (−10.6–−5.6)	0.358
Worst base excess 12–24 h	−7.9 (−10.8–−5.1)	−7.7 (−10.9–−4.9)	−8.3 (−10.6–−6.5)	0.423
Transfusion (mL)				
Packed red blood cells	1145 ± 931	1040 ± 917	1224 ± 951	0.499
Fresh frozen plasma	688 (352–1162)	381 (332–747)	925 (526–1264)	0.006
Length of ICU stay (days)	23 (16–36)	20 (15–29)	32 (18–40)	0.007
Length of hospital stay (days)	46(31–95)	44(31–94)	53(28–95)	0.982
Occurrence of MOF (day)	-	-	5.3 ± 4.0	-
Mechanical ventilation	81 (84.4%)	38 (76.0%)	43 (93.5%)	0.024
Vasopressor use	33 (34.4%)	9 (18.0%)	24 (52.2%)	<0.001
Mortality	14 (14.6%)	1 (2.0%)	13 (28.2%)	<0.001

MOF: multiple organ failure; ISS: injury severity score; AIS: abbreviated injury scale; CT: computed tomography; PT-INR: prothrombin time international normalized ratio; ICU: intensive care unit. * Maximal SOFA measured within 48 h of ED admission. ^†^ Normal reference range of PT-INR: 0.92–1.13.

**Table 3:** Type of major surgery and interventional treatment.

	Total (n = 96)	Non-MOF Group (n = 50)	MOF Group (n = 46)	*p* Value
Exploratory laparotomy *	16 (16.6%)	8 (16.0%)	8 (17.4%)	1.000
Interventional angiography ^†^	2 (2.0%)	0 (0%)	2 (4.3%)	0.227
Exploratory thoracotomy and lung repair	3 (3.1%)	0 (0%)	3 (6.5%)	0.106
Closed thoracostomy	15 (15.6%)	11 (22.0%)	4 (8.6%)	0.094
Decompressive craniectomy and hematoma evacuation	20 (20.8%)	10 (20.0%)	10 (21.7%)	1.000
Drainage catheter insertion for intracranial hemorrhage	4 (4.1%)	2 (4.0%)	2 (4.3%)	1.000
Open reduction and internal fixation (injured extremity)	5 (5.2%)	4 (8.0%)	1 (2.1%)	0.364
Limb amputation	1 (1.0%)	0 (0%)	1 (2.1%)	0.479
Conservative management	33 (34.4%)	16 (32.0%)	17 (36.9%)	0.670

* Individual name of laparotomy as follows: bleeder ligation of mesentery, esophagus repair, primary repair of ascending colon serosa, primary repair of liver laceration, wedge resection of liver, distal pancreatosplenectomy, segment resection of duodenum with gastrojejunostomy, splenectomy, segmental resection of jejunum, primary repair of injured inferior vena cava, common iliac artery, superior mesenteric vein, primary repair of diaphragm, nephrectomy, etc. ^†^ Embolization of hepatic artery.

## Data Availability

The datasets generated and/or analyzed during the current study are not publicly available due to privacy and ethical considerations but are available from the corresponding author on reasonable request.

## Supplementary Materials

The following supporting information can be downloaded at: https://www.mdpi.com/article/10.3390/jcm14103447/s1, Figure S1: Comparison of consecutive myeloperoxidase index (MPXI) for two weeks in severe trauma patients between those with multiple organ failure and those without multiple organ failure. Figure S2: Comparison of consecutive delta neutrophil index (DNI) for two weeks in severe trauma patients between those with multiple organ failure and those without multiple organ failure.

## Abbreviations

The following abbreviations are used in this manuscript:

MOF	Multiple organ failure
ICU	Intensive care unit
SIRS	Systemic inflammatory response syndrome
SOFA	Sequential organ failure assessment
MPXI	Myeloperoxidase index
DNI	Delta neutrophil index
MPO	Myeloperoxidase
MAP	Mean arterial pressure
