# Supplementary material for: Early Evaluation of Myeloperoxidase and Delta Neutrophil Indices Is Similar to 48 h Sequential Organ Failure Assessment Score for Predicting Multiple Organ Failure After Trauma
Source: J Clin Med. 2025 May 15;14(10):3447. doi: 10.3390/jcm14103447 (PMC12111808; doi:10.3390/jcm14103447)
Supplement: Supplementary file 1 [file jcm-14-03447-s001.zip › jcm-3594588-supplementary.pdf]

Supplementary Materials

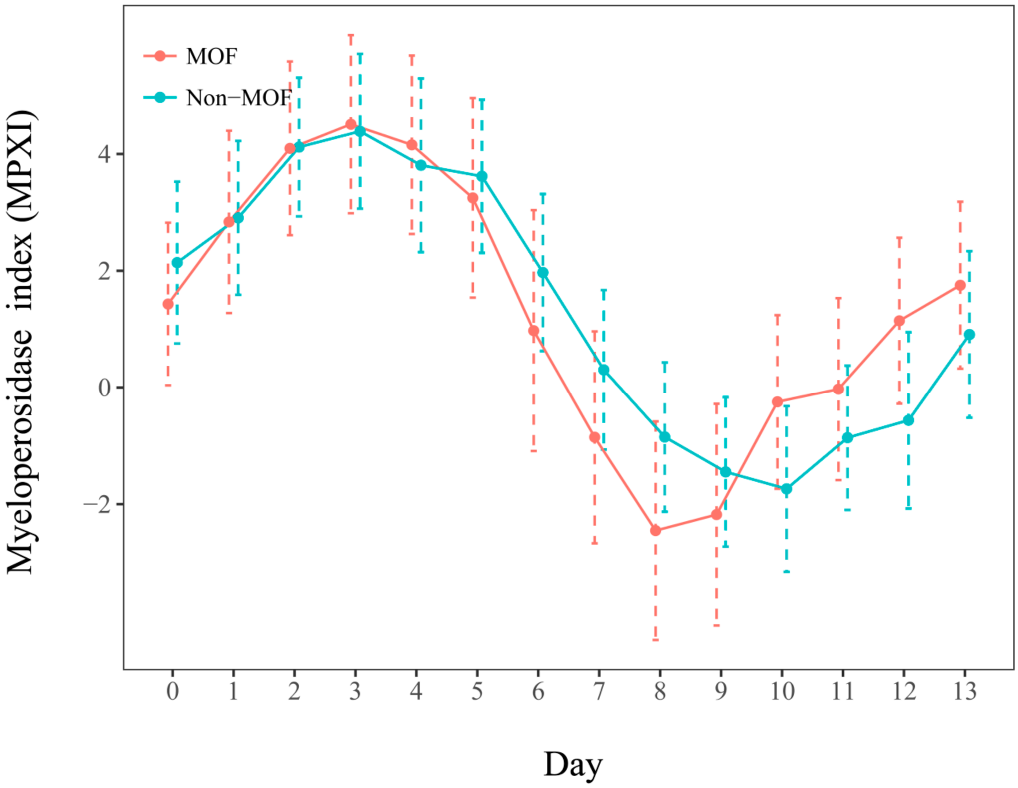

Figure S1. Comparison of consecutive myeloperoxidase index (MPXI) for two weeks in severe trauma patients between those with multiple organ failure and those without multiple organ failure.

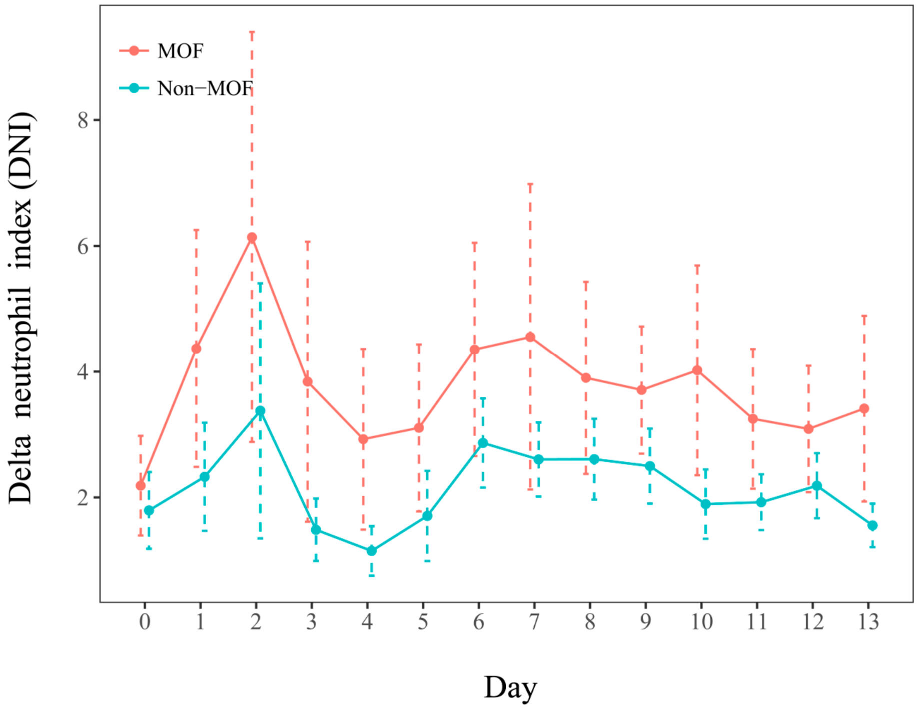

Figure S2. Comparison of consecutive delta neutrophil index (DNI) for two weeks in severe trauma patients between those with multiple organ failure and those without multiple organ failure.
